# “Fusion and binding inhibition” key target for HIV-1 treatment and pre-exposure prophylaxis: targets, drug delivery and nanotechnology approaches

**DOI:** 10.1080/10717544.2016.1228717

**Published:** 2017-02-26

**Authors:** Tanushree Malik, Gaurav Chauhan, Goutam Rath, R. S. R. Murthy, Amit K. Goyal

**Affiliations:** 1 DBT Lab, Indo Soviet Friendship College of Pharmacy, Moga, India and; 2 Centre for Nanosciences, Department of Chemical Engineering, Indian Institute of Technology Kanpur, Kanpur, India

**Keywords:** HIV transmission, fusion inhibition, targets, drug delivery, nanotechnology

## Abstract

More than 35 million people are living with HIV worldwide with approximately 2.3 million new infections per year. Cascade of events (cell entry, virus replication, assembly and release of newly formed virions) is involved in the HIV-1 transmission process. Every single step offers a potential therapeutic strategy to halt this progression and HIV fusion into the human host cell is one such stage. Controlling the initial event of HIV-1 transmission is the best way to control its dissemination especially when prophylaxis is concerned. Action is required either on the HIV’s or host’s cell surface which is logically more rational when compared with other intracellular acting moieties. Aim of this manuscript is to detail the significance and current strategies to halt this initial step, thus blocking the entry of HIV-1 for further infection. Both HIV-1 and the possible host cell’s receptors/co-receptors are under focus while specifying the targets available for inhibiting this fusion. Current and under investigation moieties are categorized based on their versatile mechanisms. Advanced drug delivery and nanotechnology approaches present a key tool to exploit the therapeutic potential in a boosted way. Current drug delivery and the impact of nanotechnology in potentiating this strategy are detailed.

## Introduction

HIV infections are considered to be the global threat of present era. A huge percentage of human community is influenced by the direct as well as related impacts of this pandemic. Antiretrovirals are currently the best option for prolonged and maximal viral suppression (Hammer et al., 2008). Currently used antiretroviral drug classes include reverse transcriptase inhibitors (RTIs), protease inhibitors (PIs), entry inhibitors (CCR5 antagonists and fusion inhibitors) and integrase inhibitors (das Neves et al., 2010a). Since no curative therapy is available, prevention is a cornerstone in the battle against HIV/AIDS, around 30 individual drugs and ﬁxed-dose combinations are available to manage this pandemic. In context to HIV prophylaxis, current research period is able to develop an overall potency of 39% with topical tenofovir (RTI) gel (Galvin & Cohen, 2004; Karim & Baxter, 2013).

Bulk of efforts has been invested to select an effective and specific target for both preventive and treatment therapeutics. Targeting the entry of enveloped viruses is a very attractive strategy in this context, since the site of action is likely to be extracellular and therefore relatively accessible; this could also limit cell toxicity. All the stages of viral entry are potentially amenable to therapeutic intervention. In this review, we are focusing on inhibition strategies of binding and fusion of HIV-virus at cellular entry step. Discussing the possible sub-targets in the context of recently developed antiviral molecules certainly opens the access to newer targets and inhibitors (Nikolic et al., 2007; Teissier et al., 2011; Chauhan et al., 2013; McGowan, 2014; Montgomery, 2015).

## Factors related to virus

Description about HIV-1 membrane, membrane lipids and membrane glycoproteins is detailed in supplementary data.

## Host cell receptors and co-receptors

### Host cell receptors

Attachment of the virus to the cell surface, involves recognition and binding to specific cell surface receptors. Table 1 provides descriptive information about these receptors.

**Table 1.** Description of host cell receptors involved in HIV-1 transmission.

Receptor	Basic details	Mechanism involved
CD4 + T cells	The primary receptor for HIV-1 is CD4 to infect T cells, macrophages. These are dispersed throughout the lamina propria of the human vagina, ectocervix and endocervix.	These cells become activated when they are presented with peptide antigens by MHC (Major Histocompatibility Complex) class II molecules, which are expressed on the surface of antigen-presenting cells (APCs). Upon activation CD4 T cells undergo a series of division to produce helper T cells (TH) and memory T cells (TM). TM cells remain in reserve whereas TH cells secrete a variety of cytokines. Some of these co-express high level of both CCR5 and CXCR4 co-receptors (Veazey et al., 2003).
Dendritic cells	Dendritic cells (DC) are located just beneath the endocervical columnar epithelium.	HIV-1 envelope (Env) interacts with DCs via a number of attachment factors. The C-type lectin receptors such as DC-SIGN (Geijtenbeek et al., 2000), mannose receptor, langerin (expressed on Langerhans cells) and DCIR are some of the best-described mechanisms of HIV capture by DCs. GM3 is a glycosphingo-lipid called gangliosides promotes their specific recognition by DCs. Siglecs, a family of proteins including membrane gangliosides. Increased Siglec-1 expression on the surface of DCs, enhanced HIV uptake by mature DCs (Sedwick, 2012).
Langerhans cells (LCs)	These are a DC subtype residing within the outer squamous epithelium of the skin or mucosa. HIV-specific receptors are expressed by these LCs, including CD4, CCR5 and the C-type lectin langerin (CD207), but not CXCR4.	Antibodies that bind CD4 and CCR5 partially block the uptake of R5-tropic HIV-1 by LCs (Hladik et al., 2007). LC serves detecting HIV-1 entry into the vagina, through C-type lectin receptor (CLR), Toll-like receptor (TLR) and other recognition receptors (Chang & Altfeld, 2009). The entry by the assistance of Langerhans cell solely depends on the activation status of the LC. As immature LCs prevent HIV-1 infection by clearing invading HIV-1 through the C-type lectin langerin but blocking langerin function by high virus concentrations enables HIV-1 transmission by LCs (Nishibu et al., 2006).
Macrophages	Macrophages in the female genital tract constitutively express CCR5 during the period of activation and emigration from the mucosa.	These are professional APC, triggering antibody responses by the presentation of pathogen derived peptides via the MHC-II pathway to CD4+ T cells and activating CD8+ cytotoxic T-cells (CTL) by cross-presentation of HIV-1 antigens. Since macrophages secrete cytokines that recruit T lymphocytes to sites of infection. They can “support” establishment of viral infection by enlarging the number of primary target cells (Ackerman & Cresswell, 2004). An infected macrophage can infect at least one T cell every six hours for many weeks (Groot et al., 2008).
Galactosylceramide (GalCer)	It is now well established that HIV infection in CD4-negative cells follows some alternate routes for several cell types. Mentioning epithelial cells as target for HIV-1, the mechanism proposed for its transmission through CD4-negative cell involves a specific receptor, i.e. glycosphingolipid galactosylceramide (GalCer) (Delézay et al., 1996; Nittayananta et al., 2016).	Cell lines showed that virus uses its surface envelope glycoprotein gp120 to get access via cells of epithelial origin. HIV-1 also exploits heparan sulfate proteoglycans (HSPGs) receptors for attachment purpose.
Heparan sulfate proteoglycans (HSPGs)	These HSPGs consist of a core protein and unbranched anionic chains composed of repeating disaccharides units (sulfated uronic acid and hexosamine residues).	Mechanism involved with this receptor can be explained by the role of negatively charged sulfated groups of heparan sulfate (HS) chains in the virus attachment to the host cell surface (Lindahl, 2007; Rusnati et al., 2009).

### Host cell co-receptors

CD4 binding causes conformational changes in gp120 that binds it to a second receptor, a co-receptor. HIV-1 strains use the chemokine receptor CCR5 in conjunction with CD4 for virus entry and in absence of CCR5, CXCR4 either in place of or in addition to CCR5. Co-receptor choice is notably the V3loop and, to a lesser degree, the V1/2 region within gp120. This highly conserved region may be the target for neutralizing antibodies (NABs) (Kwong et al., 1998). The relatively strong binding to CD4 causes the joining of two β-hairpins to form the bridging sheet domain. gp120 domains rotate and move away from the central stalk, exposing the V1/V2 and V3 loops to the target membrane (Liu et al., 2008). Membrane fusion is a cooperative process of four to six CCR5 receptors, multiple CD4 molecules and three to six Env trimers are needed to form a fusion pore (Kuhmann et al., 2000). Viruses using CCR5 are largely transmitted and endure throughout infection and those that use CXCR4 often emerge later on in the course of infection and have been associated with more rapid disease progression and CD4 cell decline.

## Mode of transmission

**Figure 1.**
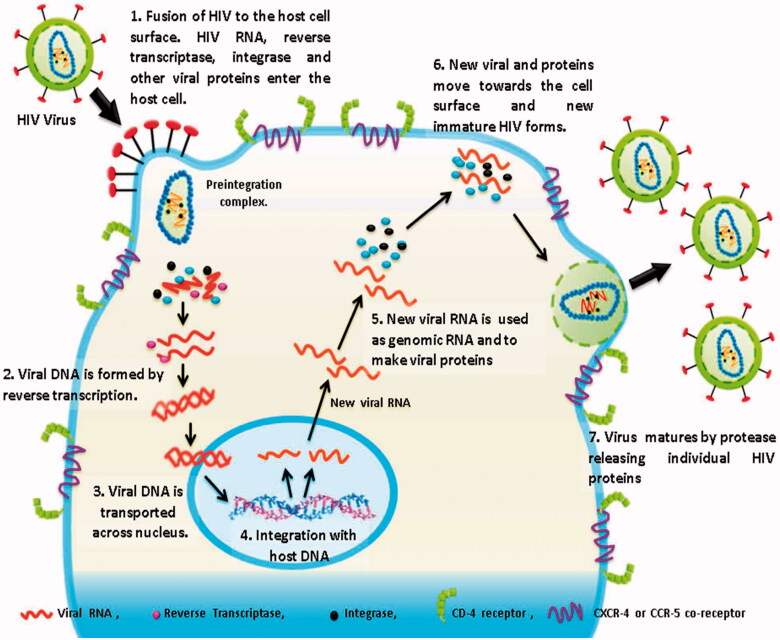
Transmission of HIV-1 virus.

Briefly describing this whole transmission process of HIV, we take this transmission as a triple phase process. First phase (P-1) is HIV invasion; second phase (P-2) is HIV replication and third is budding of newly formed virons from the host cell (Figure 1) (description about the mode of HIV-1 transmission is detailed in supplementary data).

## Targets for fusion and binding inhibition

**Figure 2.**
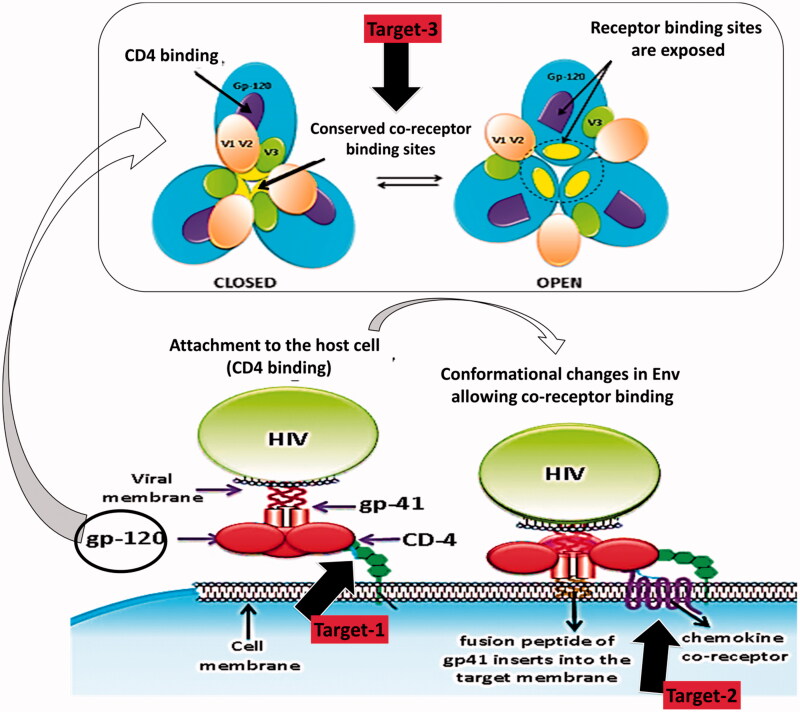
Targets for fusion and binding inhibition.

Fusion inhibitors perform extracellularly prior to assault of the host cell. So they are not liable to cellular efflux transporters that lesser the effective intracellular concentrations of additional classes of antiretrovirals. Their absence of intracellular processing may pay to their low toxicity proﬁle and reduces cross-resistance with recognized intracellular agents. With an exclusive mechanism of action they signify a new fourth class of ARVs (Greenberg & Cammack, 2004). They block the last step in the three-step viral entry process comprising of attachment, co-receptor binding and fusion (Greenberg & Cammack, 2004). Three key steps in viral entry have been embattled for drug development, i.e. inhibition of CD4 binding, inhibition of co-receptor binding and obstruction of the gp41 conformational changes that allow viral fusion. Figure 2 explains these abovementioned targets in a descriptive way. Fusion inhibitor molecules also act as peptide mimics, which block the interactions that are obligatory for HIV-1 viral entry (Wild et al., 1994).

### Inhibition of CD4 binding

The CD4 binding site (CD4BS) is at the interface of all three gp120 domains. CD4 binding site is extremely well-preserved and NABs can efficiently block this step. Tetravalent CD4-IgG and Pro542 are some examples that can bind gp120 and restrict its ability to accept the CD4-bound conformation, thus disturbing the entry process. Although CD4 is not involved in the process, CD4BS antibodies exert inhibitory activity on co-receptor binding and envelope glycoprotein mediated syncytium formation. It was also believed (not entirely) that HIV-1 envelope glycoproteins elicit NABs raised against both the variable and conserved regions of the envelope glycoproteins during natural infection (Raja et al., 2003).

### Inhibition of co-receptor binding

CCR5 is a member of a large family of G-protein-coupled, seven-membrane-spanning receptors (GPCRs). CCR5 has progressed as an attractive target for therapeutic intervention because of its cell type-specific expression and its important role as the major co-receptor for HIV-1. The CCR5 molecule does not appear essential for immune function and survival. So inhibition of CCR5 may not be accompanying with adverse effects on cellular function (Marmor et al., 2006). Chemokine receptor CCR5 and to a slighter extent CXCR4, have been under extreme targeting for small molecular weight inhibitors. This includes the approaches like prevention of co-receptor binding to gp120 through steric or allosteric hindrance, second by inducing receptor down-regulation thereby limiting the number of HIV entry points and third by the development of inhibitors for blocking the co-receptor binding site on gp120 (Kuhmann et al., 2000). Antibodies against the CCR5 N-segments also block entry with variable degrees of activity, but usually are less potent than those against the extracellular region (Lee et al., 1999). Only one potent anti-CXCR4 antibody (12G5) that inhibits HIV-1 entry has been well characterized. The 12G5 murine monoclonal recognizes an epitope in extracellular loops of CXCR4 (Phogat et al., 2007). The chemokine variants also competitively block interaction of gp120 with the chemokine receptors (Matos et al., 2010). Chemokines bind to their receptors at two positions, one in the NT and the other within the TM domain. The binding sites on CXCR4 have been restricted to the extracellular domain (Donzella et al., 1998).

### gp41 targeting

A prominent pocket on the surface of a central trimeric coiled coil within gp41 is a potential target for inhibiting HIV-1 entry. In gp41, a central three stranded coiled coil formed by the N-terminal regions of gp41 is surrounded by helices derived from the C-terminal end of the gp41 ectodomains (Eckert et al., 1999). Peptides corresponding to these regions of gp41 are known as N-peptides and C-peptides (Kahle et al., 2009). The principal targets for gp41 inhibitors are two heptad repeat (HR) fragments in the N- and C-terminal regions of the gp41 ectodomain symbolized N-HR and C-HR, respectively (Lu et al., 1995). Synthetic C-peptides act in a dominant-negative manner by binding to the transiently exposed coiled-coil N-peptide region in the prehairpin intermediate. Therapeutic agent that goals this gp41 pocket would likely be relatively elusive to the emergence of resistant viral strains (Chan et al., 1998). However, C-peptides not containing pocket-binding residues, such as T-20, are more vulnerable to the emergence of resistant virus than C-peptides containing pocket-binding residues, such as T649 (Rimsky et al., 1998).

The peptide inhibitor efforts to bind in the trimeric coiled loop of gp41, performs a dominant-negative mechanism (Weissenhorn et al., 1997). C peptides perform at this intermediate phase, subsequently the native state has undertaken a conformational transition but before establishment of the hairpin structure since, once this gp41 core is accumulated, it is extremely stable and the melting temperature of the gp41 core is in surplus of 90 °C which is unlikely to be interrupted by exogenous peptides (Matos et al., 2010). Consistent using this vision, the C peptide DP178 binds to gp41 only after collaboration of the envelope complex with cellular receptors (Furuta et al., 1998).

Structurally mimicking engineered proteins of all or part of the N-HR coiled coil can prevent HIV-1 entry by binding the gp41 C-HR regions (Eckert & Kim, 2001; Louis et al., 2001). A well-considered example is the 5-helix protein that contains all three N-HR sections but only two C-HR segments; when suitably folded, 5-helix exposes a single C peptide binding spot that strongly interacts with gp41 C-HR fragment (Root et al., 2001). 5-Helix and C-peptides do not interact with the native state of Env earlier to gp120/CD4 interaction (Melikyan et al., 2000).

Effects of first and second generation peptide inhibitors in inhibiting conformational changes necessary for viral fusion provide a proof of principle that the entry process is a viable target for therapeutic intervention. Molecules that block TOH (trimers of hairpins) formation can effectively inhibit HIV-1 membrane fusion both *in vitro* and *in vivo* (Kahle et al., 2009). Inhibitors bind the N-HR or C-HR section prior to bundle creation and prevent folding of gp41 into its TOH conformation (Root & Steger, 2004). The broad inhibitory activity of C peptides against diverse HIV isolates is explained by the highly conserved nature of the hydrophobic groove to which these peptides bind (Chan et al., 1997). C-peptides goal the N-HR in its coiled-coil form, binding the same hydrophobic indentations that would normally interact with gp41 CHR divisions (Kilgore et al., 2003). Several peptides that mimic the sequence of the C- and N-helical regions of HIV-1 have been found to inhibit fusion either by blocking the interaction between the N- and C-helical regions or by promoting the dissociation of gp41 trimers into monomers and thus preventing the formation of the 6-helix bundle fusogenic state of gp41 (Weissenhorn et al., 1999).

## Present therapeutics and prophylaxis

### CD4–gp120 attachment inhibitors

Attachment inhibitors like BMS-043 and BMS-806, PRO 2000, TMB-355 or Ibalizumab and PRO 542, are designed to inhibit the binding of gp120 to the CD4 receptor and are in pre-clinical or clinical development phase. These act extracellular prior to viral cell invasion but fewer like TMB-355 targets the essential host CD4 receptor instead of a speciﬁc viral target, which may result in undesirable side effects.

PRO-542 (CD4–IgG2) is a tetravalent soluble recombinant antibody-like fusion protein that includes four replicas of the virus binding CD4 domain (Allaway et al., 1995) and mimics the CD4 receptor. It is one of the gp120–CD4 binding inhibitors in more progressive phases of clinical development. Phase I studies determined that PRO-542 was well tolerated without apparent dose-limiting toxicities (Jacobson et al., 2000). Besides, pre-clinical studies uniting PRO-542 and enfuvirtide have recommended synergy in the inhibition of HIV replication (Nagashima et al., 2001). TMB-355 is a non-immunosuppressive monoclonal antibody focused against the CD4 receptor to compete with HIV gp120 for CD4 binding. Therefore, TMB-355 might inhibit post-viral binding conformational changes which are essential for the successful entry of HIV into the cell. cyclotriazadisulfonamide (CADA) compounds, down-regulate the cellular receptor, CD4. Some synthesized analogs of CADA proved to be highly effective in decreasing cellular CD4 and in acting as HIV entry inhibitors (Vermeire et al., 2002). CADA shows specific inhibition of the CD4–gp120 binding by assuming down-regulation of the CD4 receptor expression at the post-translational level (Vermeire et al., 2003). BMS-378806 (a prototype small molecule attachment inhibitor) is a newly exposed molecule with high affinity to prevent the conformational changes induced in gp120 after CD4 binding. Binding of BMS-806 to HIV gp120 is highly specific, reversible and co-receptor independent. This compound exhibits potent inhibitory activity against a panel of R5, X4 and R5/X4 HIV-1 (Lin et al., 2003). The study data shows that BMS-378806 is a representative of a new class of HIV inhibitors having the potential to become a valued addition to our current antiretroviral drugs (Lin et al., 2003). BMS-663068 has revealed effective antiviral activity in early phase studies and phase 2b trials are at present in progress (Henrich & Kuritzkes, 2013).

Synthetic compound 2-aminothiazolones evolved as novel anti-HIV agents. It acts at the very early stage of the HIV-1 entry process through inhibition of the gp120–CD4 protein–protein interaction (Tiberi et al., 2014). Synthetic compound pyrimidinediones signify high efficacy and potency against HIV-1 with a dual mechanism of antiviral action including both virus entry and reverse transcription (Watson Buckheit et al., 2011). Cyanovirin-N (CV-N) is a cyanobacterial protein having potent activity against HIV (Dey et al., 2000). CV-N directly goals HIV spike glycoproteins, preventing attachment and consequent viral fusion, and successfully renders the virus non-infectious (Shenoy et al., 2001). This comprises several binding to high-mannose oligosaccharides mainly in the C2–C4 region of the gp120 protein (Hu et al., 2007). Then it blocks the conformational changes essential for virus–target cell attachment and subsequent fusion (Buffa et al., 2009). CV-N has broad-spectrum antiviral activity, both for numerous steps in the HIV entry mechanism (Dey et al., 2000). CV-N has high binding afﬁnity and nonexistence of toxicity (Balzarini et al., 2006). The green tea flavonoid, epigallocatechingallate (EGCG), anticipated having an anti-HIV-1 effect also high affinity to CD4 molecule for blocking the binding of gp120 to the CD4 molecule on T cells (Williamson et al., 2006).

### CCR5 and CXCR4 antagonists

CCR5 and CXCR4 antagonists are divided into groups depending on their size. Large molecules, such as PRO-140, or molecules with a medium size, as Met-RANTES and AOP-RANTES, which are modified CCR5 natural ligands, make CCR5 inaccessible. Finally, several small-molecule inhibitors directed against CCR5 (TAK-779, SCH-C, SCH-D, UK427, 857 and GW-873140) or CXCR4 (KRH1636) are in several stages of clinical development phase. Most CCR5 antagonists are small molecules which block the gp120–CCR5 interaction after binding to the co-receptor. These also cause undesirable side effects, since these are target essential host molecules, rather than speciﬁc viral targets (Greenberg & Cammack, 2004). Maraviroc (MRV) (UK-427, 857) is a CCR5 antagonist. It attached to the transmembrane co-receptor cavity, within the 2, 3, 6 and 7 helix, that is different from the region targeted by TAK-779. The drug is presently in innovative steps of clinical development and is anticipated to be approved in the near future (Briz et al., 2006; van Lelyveld et al., 2015). Studies implemented with Aplaviroc (GW-873140) and other CCR5 antagonists (SCH-C and TAK-779) showed that they exhibit potent activity against R5 viruses. Aplaviroc act directly with second extracellular loops (ECL2) and not with the transmembrane cavity (Maeda et al., 2004).

The TAK-779 is a small molecule which acts at the membrane fusion stage by obstructing the interaction of gp120 with co-receptor CCR5. The binding site for TAK-779 is on ECL of CCR5. TAK-779 also avoids the binding of the ordinary CCR5 ligand, RANTES. It is not in clinical development phase but TAK-652 is under clinical development. PRO-140 is a monoclonal antibody focused against the CCR5 co-receptor, to inhibit the binding of HIV gp120. Cenicriviroc is a small-molecule CCR5 antagonist arrived in phase 2b studies. The potent antiviral activity of AMD3100 against X4 strains has been established in different *in vitro* and *in vivo* studies (but it is not further developed for HIV therapy because of its poor bioavailability severe side effects) (Donzella et al., 1998; Flomenberg et al., 2005). KRH-1636 is another CXCR4 antagonist, with antiviral activity similar to that of AMD3100. KRH-2731 is a novel CXCR4 antagonist (Ichiyama et al., 2003). Phase II trials are currently continuing. The binding site for CXCR4 antagonists is positioned in the ECL2 of the CXCR4 co-receptor (Murakami et al., 1997; Labrosse et al., 1998). Due to high negative charge on the surface of CXCR4, the interactions with the HIV gp120 V3 loop are electrostatic. HIV strains using CCR5 co-receptors are inhibited by the CCR5 ligands RANTES, MIP-1a and MIP-1b; correspondingly strains using CXCR4 co-receptor are inhibited by the CXCR4 ligand SDF (stromal-derived factor). Small molecule inhibitors alike ALX40-4C and T22 prevent entry of HIV strains that make use of CXCR4, thereby blocking its interaction with gp120 (Matos et al., 2010).

### Fusion inhibitors

Enfuvirtide is the ﬁrst in this category to reach market approval. Enfuvirtide has a unique mechanism of action, high viral target speciﬁcity, high efﬁcacy and low toxicity. Enfuvirtide is a peptide mimetic (Greenberg & Cammack, 2004). It is homologous to a segment of the HR2 region of gp41 corresponding to amino acids 643–678. Enfuvirtide obstructs the HR2 binding site on the gp41 trimeric coiled coil assembly, thereby inhibiting the development of the hairpin structure and subsequently, the fusion (Matthews et al., 2004). Enfuvirtide is previously known as T-20 and DP-178 would drag to the opposite NHR (Matos et al., 2010). T20 (enfuvirtide) C-peptide, efficiently suppress HIV-1 infection in human (Kahle et al., 2009). Second generation of peptide inhibitors symbolizes T-1249, it fixes to gp41 and avoids its fusogenic conformation, hindering viral entry into host cells (Fatkenheuer et al., 2005). This agent is a synthetic compound utilizing non-native sequences corresponding to the HR2 region and has been shown to inhibit enfuvirtide-resistant virus. Development of drug resistance in first generation (T20) and drug formulation problem in second generation (T-1249) halted their further clinical development. Third generation fusion inhibitors in the form of sifuvirtide are as initially designed on the basis of three-dimensional structural information of gp41. It has been assessed by the phase I clinical trials and is presently in phase II clinical studies. It was recently demonstrated that the M-T hook structure can be used to design a short CHR peptide that specifically targets the conserved gp41 pocket rather than the T20-resistant sites. Recently an attempt was made to develop more potent HIV-1 fusion inhibitors using multiple biophysical and functional approaches. HP23 (a 23-residue peptide), following M-T hook structure and pocket-binding sequence, had significantly improved anti HIV-1 activity (including on T20 and MT-SC22EK resistant HIV-1 mutants). It was considered as the most potent inhibitor of both M-T hook-modified and unmodified control peptides.

5-Helix binds the C-peptide region of gp41 and acts by sequestering one of the helices necessary for the formation of the six-helix bundle protein. Peptide mimetics fusion inhibitors T-649 having homology with the HR2 region of gp41 inhibit hairpin structure formation in a parallel way to enfuvirtide. C34 peptide has homology to a region which creates the six-helix bundle and blocks its formation. A cyclic molecule D-peptide (DP-107) is homologous to HR1 that binds HR2 (a pocket region within the six-helix structure) (Root et al., 2001; Koshiba & Chan, 2003). “RPR103611” a non-peptide triterpene compound disrupts the association of gp120–gp41 in CXCR4-tropic viruses by targeting the loop region of the gp41 leucine zipper (Greenberg & Cammack, 2004).

C-helical peptides such as C34 blocks fusion through binding to the hydrophobic grooves that line the interior N-terminal trimeric coiled coil core of the gp41 ectodomain (Lu et al., 1995). N-helical-derived peptides such as N36 are much less operative inhibitors. They goal the N-helical region of the prehairpin intermediate by making fusion-incompetent heterotrimers (Weissenhorn et al., 1999). Binding of HIV-1 envelope glycoprotein (Env) to its cellular receptors provokes a variety of signaling events, comprising the activation of selected tyrosine kinases (Stantchev et al., 2007). Genistein prevents infection of macrophages made by Env glycoprotein. This inhibitory effect of genistein implied prevention of the virus entry process (Tobiume et al., 2003; Cavrois et al., 2004).

#### Lectins

Several lectins having strong-mannose carbohydrates binding site on the surface of virus envelopes have been found to have antiviral activity. Some specific algal lectins such as Cyanovirin-N, Griffithsin, Microcystisviridislectin, Scytovirin, Oscillatoriaagardhii agglutinin, demonstrate high anti-HIV activity (Lin et al., 2003). Lectin actinohivin (AH), exhibits potent *in vitro* anti-HIV activity by interacting to high-mannose type glycans (HMTGs) of highly glycosylated gp120 of HIV. Griffithsin (GRFT), a 12.7 kDa carbohydrate-binding protein, is highly potent inhibitor of both CXCR4 and CCR5-tropic viruses of HIV-1. GRFT also blocks cell fusion and cell-to-cell transmission of HIV (Doms & Trono, 2000). Griffthsin with tenofovir, MRV and enfuvirtide gives synergistic activity proﬁle. GRFT inhibits viral replication at primary levels (Emau et al., 2007). GRFT efficiently prevents DC-SIGN-mediated transfer of HIV-1 to CD4+ T-lymphocytes and capable of expelling gp120 from the gp120–DC-SIGN complex.

## Drug delivery

Disappointing results of the current approaches, demand smart drug delivery approaches with better delivery, superior safety profiles and potential for improved patient compliance. Challenge to design an efficient delivery system is a smart task. This designing contest requires a need based approach where different delivery routes have their specific requirement. Localized drug delivery is very much explored in this case, as it offers several additional advantages when compared to other routes. Now, the dosage from designing approach must be entirely different in every category. Specifically for topical purpose; gels, creams, tablets suppositories are among the conventional formulations and vaginal rings are most common for long term controlled delivery (Goyal et al., 2015; Gaurav et al., 2015b; D'Cruz & Uckun, 2014). In this specific case bioavailability achieved is affected by numerous physiological factors, and the ability of the formulation to effectively deliver drug molecule may vary with the menstrual cycle, pH variations and the presence of co-pathogens. There is a special emphasis on mucoadhesive gel systems for the delivery of intravaginal microbicide. The remarkable elastic character and the improved rheological properties of the mucoadhesive gel prolong the residence time at the application site. Selection of correct viscosity of the formulation is important in order to provide adequate retention and distribution in the vagina. A list of selected polymers is mentioned in Table 2.

**Table 2.** List of polymers used for the formulation of mucoadhesive vaginal gel formulation system.

Polymer	Properties	Market products
Polycarbophil	Low irritation and toxicity, non-sensitizing, good stability, eliminate the effect of vaginal discharge which shortens the residence time of vaginal formulations.	Miphil (acid buffer), Replens® (with carbopol 974p)
Carbopol (974p, 934p, 940p)	Good buffering capacity (around pH 4), nontoxic and nonirritating, Vaginal fluid’s dilution may provide better wetting of polymer and hence formation of stronger mucoadhesive bonds and prolongation of the residence time.	Miphil (acid buffer), Advantage-S® (Carbopol 974p with polycarbophil), Replens® (Carbopol 974p with polycarbophil)
Carrageenan	Combination of (kappa and lambda) has proved topical microbicide, relatively nontoxic and nonirritating, for topical application, Kappa carrageenan also acts as absorption inhibitor.	Carraguard™, PC-815 gel combining Carrageenan with MIV-150, PC-515 gel
Chitosan	Nontoxic and nonirritating, biocompatible with both healthy and infected skin, useful as a carrier for longer release.	Chitosan based metranidazol vaginal mucoadhesive gel
Cellulose derivatives HEC, HPC, HPMC, Na-CMC	A wide versatility of mucoadhesion, and viscosity ranges is available. Nontoxic and nonirritant.	Conceptrol® (Na-CMC), K-Y® (HEC), Gynol-II, (Na-CMC), Ushercell (HEC), Monocaprin hydrogel (HPMC)
Sodium-alginate	Compatible for vaginal use, and already tried for N-9 formulation	Delivery gels were alginate crosslinked with calcium chloride containing 3% N-9 and were manufactured over a pH range of 3.4–5.9
Polyacrylic acid	These polymers are the most investigated bioadhesive polymers for vaginal applications	BufferGel™, Acidform™ gel
Pectin, gum acacia and tragacanth	Nontoxic, nonirritating and already used in vaginal formulation	Aci-Jel (gum acacia and tragacanth)

Nanotechnology has its own role to play when considered for this task. The small size of the nanocarriers allows them to be transported more easily across the mucosal barriers by passive or active transport and possess the host targeting ability after entering the blood stream (Mallipeddi & Rohan, 2010b; Date & Destache, 2013). Essential properties of different nanosystems such as size, particle shape and surface charge can modify its bioavailability and targeting. In case of active targeting, a suitable surface modification “most frequently by surface attachment of speciﬁc ligands” is capable of identifying target cells or sites (das Neves et al., 2010b; Gaurav et al., 2015b). Enfuvirtide and MRV therapies in a conventional solid dosage form (tablet and capsules) have been tried and still under surveillance. Poor *in vivo* performance after a successful *in vitro* screening points towards the poorly managed drug delivery. T20 and sifuvirtide are also delivered by subcutaneous injection but a huge difference in half-life was observed for both these candidates. One important limitation of many current antiretroviral drugs is their unavailability to circumvent efflux pumps (particularly P-glycoprotein) that are present, in the membrane of several HIV-target cells. An effective delivery of such drugs requires an ideal platform and nanotechnology based approaches are a good area to watch out.

## Nanotechnology approaches

**Figure 3.**
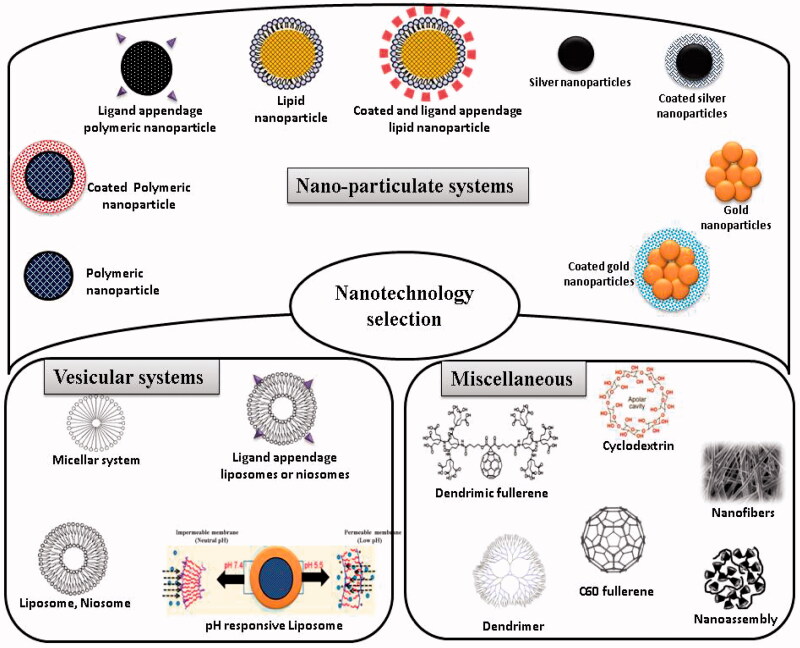
Nanotechnology tools that can be exploited for the rational delivery of fusion inhibitors.

General properties of nanosystems that favor their use in antiretroviral drug delivery are well known and include versatility (virtually drugs may be encapsulated), good toxicity proﬁle, possibility of drug-release modulation, high drug payloads, relative low cost, easiness to produce and possible scale-up to mass production scale (Vyas et al., 2006; Mallipeddi & Rohan, 2010a; Gaurav et al., 2015b,c). Nanocarriers provide an option for the optimal and controlled drug delivery at the target sites in the body. The small size of the nanocarriers enables them to penetrate through cells and deliver drugs intracellular without risking extracellular degradation (Mallipeddi & Rohan, 2010b). Conversion of pure drug to nano-scale particles leads to dramatic increase in the surface area and dissolution kinetics leading to increase in bioavailability and reduction in pharmacokinetic variability (Patravale & Kulkarni, 2004; Desai et al., 2012). Moreover, their ability to incorporate, protect and/or promote the absorption of non-orally administrable anti-HIV drugs, namely mono- or oligonucleotides is of importance to improve the bioavailability of several molecules (Leroux et al., 1996; De Jaeghere et al., 2000; Gaurav et al., 2015c). Some properties of these nanotechnology-based systems, e.g. prolonged release of active agents and ability to penetrate epithelial linings, are important advantages that may favor their utilization in the ﬁeld of microbicides. Figure 3 mentions a compilation of the nanosystems that are currently under research or can be used in future.

### Vesicular systems

This delivery system can be explained as a highly ordered association of one or more concentric lipid bilayers for delivery of drug to the site of infection, preventing drug degradation with no adverse effects (Kumar et al., 2011; Chen et al., 2015; Goyal et al., 2015). The encapsulation of the drug in the vesicular structures prolongs the survival of the drug in systemic circulation, thus reduces the toxicity. Bioavailability of poorly soluble drug increases thereby decreasing the cost of therapy by encapsulating the drug in vesicular delivery system. They can incorporate both lipophilic as well as hydrophilic drugs. Vesicular systems increase the bioavailability of the poorly available drugs. Drug selectively reaches to the site of infection and remains in the systemic circulation for longer time to produce effective results without causing toxicity. The elimination time of rapidly metabolizable drugs increases by its encapsulation in vesicular system thereby causing sustained release effect. Various novel approaches have been developed and are under development focusing different routes of administration to attain sustained, controlled and targeted drug delivery (das Neves et al., 2016). A liposomal system Novasomes® was used to deliver 2 RANTES (a truncated fragment of RANTES presenting high specificity for CCR5) binding via vaginal route. Novasomes® 7474 are 200–700 nm non-phospholipidic liposomes comprising monoesters of PEO fatty acids, cholesterol and free fatty acids (Kish-Catalone et al., 2007). Native activity of −2 RANTES was effectively restored in this liposomal formulation where only mild signs of inflammation were seen during *in vivo* studies. This liposomal formulation also showed an efficient pre-exposure prophylaxis when applied (−2 RANTES/liposome complexes in PBS) 30 min before challenge in cynomolgus macaques. Cationic liposomes were successfully developed for the delivery of fusion inhibitor sifuvirtide. Strong electrostatic interactions were studied between sifuvirtide with cationic phosphoplipids providing a great platform for the delivery of anionic peptide sifuvirtide with improved stability and efficacy (Franquelim et al., 2011). Earlier in an attempt to develop safe, effective and stable intravaginal microbicides a large multilamellar liposomal formulation (in ointment base) was developed. These liposomes comprising cardiolipin (an anti-HIV lipid) significantly inhibited HIV infections, where reports suggest that cardiolipin composition affect the rate and extent of HIV-1 fusion (Malavia et al., 2011).

### Polymeric and lipid based nanoparticles

Polymeric nanoparticles range between 10 and 1000 nm, catch great attention while delivering various anti-HIV moieties via different routes (Corsi et al., 2016; Narasimhan et al., 2016). Owing to several properties possessed by these systems such as easy to synthesize, biocompatible, biodegradable, non-immunogenic, inexpensive, nontoxic, etc., they are considered as versatile nanosystems to study. Different polymers are such as poly(lactic-co-glycolic acid) (PLGA), chitosan, poly(actic acid) (PLA), polyethylene glycol (PEG), poly(γ-glutamic acid) (γ-PGA) and polylysine (PLL). Polymeric nanoparticles can (1) protect encapsulated active molecule from in local biological degradation, (2) yield sustained and enhanced cross-presentation of the active moiety to the viral as well as host cell, (3) undergo endolysosomal escape after uptake by immune cells; and (4) can be tailored by various methods to target specific site (either viral or host cell receptor) (Peek et al., 2008; Hamdy et al., 2011; Qu et al., 2016).

Lipid nanoparticles are particles of nanosize range 50–500 nm made up of lipids that are solid at body temperature. They have potential for sustained/controlled and targeted drug delivery so preferred for pharmaceutical application. Types of lipid nanoparticles were considered for medicinal use. Solid lipid nanoparticles (SLN) have ability to incorporate a wide variety of drugs. These are the spherical shaped particles made up of biodegradable solid lipids and emulsifiers that protects chemically labile ingredients against decomposition (Zhang et al., 2007; Elsabahy & Wooley, 2012). Nanolipid carriers (NLC) consist of solid lipid matrix with a liquid lipid phase content that enhances greater drug encapsulation and drug loading due to long term stability. SLN and NLCs because of its inherent properties can accommodate both hydrophilic and lipophilic drugs. Further the presence of amphiphilic lipids and emulsifiers makes lipid nanoparticles a suitable alternative to delivery of BCS II and IV (Müllertz et al., 2010) drugs.

SLNs are composed of low cost and biodegradable solid lipid (Pardeike et al., 2009). Sustained drug release and site specificity for drug delivery can be achieved by altering the properties of lipid based nanocarriers, such as their lipid composition, size and surface charge. The presence of liquid lipid in the NLC confers long-term colloidal stability and greater drug encapsulation and loading unlike SLN phospholipids such as cardiolipin has shown ability to inhibit HIV-1 *in vitro*.

Polymeric nanoparticles are amongst the trial candidates gaining focus for their potential delivery of fusion inhibitors. Poly(lactic-co-glycolic acid), PLGA nanoparticles encapsulating PSC-RANTES revealed significant anti-HIV activity in cell cultures when compared with unformulated PSC RANTES (Ham et al., 2009; Fumakia et al., 2016). An attempt was made for the co-delivery of HIV-1 entry inhibitor and nonnucleoside RTI shuttled by nanoparticles. Biodegradable polymeric nanoparticles were synthesized to encapsulate nonnucleoside RTI (NNRTI) DAAN-14f (14f), surface-conjugated with HIV-1 fusion inhibitor T1144, designated T1144-NP-DAAN-14f (T1144-NP-14f) and aiming to achieve enhanced cellular uptake, improved antiviral activity and prolonged blood circulation time (Li et al., 2016).

### Metal nanoparticles

Metallic nanoparticles are interesting options owing to their intrinsic therapeutic potential and drug carrying properties. Exceptional surface property and smaller size (2–40 nm) provides ample opportunity to functionalize their surface and attach a selective therapeutic moiety. Inorganic metals such as antimony, iron, platinum, calcium, gold and silver have long history as therapeutic agents. Infection inhibition could be enhanced by altering the nanoparticle diameter and/or physical properties and the density of conjugates on the nanoparticle surface (Gaurav et al., 2015b).

Gold nanoparticles are effective HIV-1 fusion inhibitors. Gold nanoparticles stabilized with PEG were shown to inhibit M-tropic, T-tropic, dual tropic and resistant isolates of HIV-1. They prevent the viral entry by binding with gp120 and inhibit CD4 attachment (Vijayakumar & Ganesan, 2012). Gold nanoparticles layered with numerous copies of an amphiphilic sulfate-ended ligand are able to bind the HIV envelope gp120 to prevent HIV infection of T-cells (Di Gianvincenzo et al., 2010). Gold-based compounds have shown favorable activity against HIV-1. A typical example is auranofin which resulted in an elevated CD4+ T-cell count in an HIV patient being treated for psoriatic arthritis (Fonteh et al., 2010).

Silver nanoparticles (AgNPs) and NABs target both envelope gp120 and gp41. The addition of AgNPs to NABs exerts an additive effect and highly neutralizing potency to prevent cell-associated HIV-1 infection (Lara et al., 2011). Silver nanoparticles show anti-HIV activity at primary stage of viral replication, as a virucidal agent or as an inhibitor of viral entry. They get attached to gp120 and prevent CD4-dependent virion binding, fusion and infectivity. Also both silver sulfadiazine (AgSD) and silver nitrate (AgNO) salts, show anti-HIV activity. Silver nanoparticles interact with the two disulfide bonds situated in the carboxyl half of the HIV-1 gp120 glycoprotein (Lekutis et al., 1992) and modify this viral protein by denaturing its disulfide-bonded domain (Lara et al., 2010). PVP-coated nanoparticles synthesized by consuming glycerin as both reducing agent and solvent have shown favorable activity against HIV (Elechiguerra et al., 2005).

### Dendrimers

The word dendrimers itself defines the class of macromolecules as novel, highly branched having 3D architecture differentiating it from linear polymers (Poxon et al., 1996). Dendrimers word can also be described as the combo of two words, “Dendron” means Tree and “mer” meaning branching. Thus it is a tree like structure characterized by multiple layers of interior branching from central core, which forms repetitive addition of branched units to its central core molecule by step-growth polymerization process. Dendrimers are very effective against viral infection caused by especially HIV and Herpes virus, thus having good antiviral/antibacterial properties. The key factors behind its antiviral/antibacterial properties are surface charge property, its 3D structure and the effective size of dendrimer. The conformational changes occur during branching of the dendrimer molecule which directly affects the viscosity of the molecule. The generation number of dendrimers relates viscosity parameter of dendrimers. As generation number increases, the viscosity increases to a maximum and then decreases at higher molecular weight causing change in conformation at maximum viscosity and exerts a bell-shaped viscosity curve. Thus higher molecular weight and high generation dendrimers are not very viscous thus easy to handle as compared to other linear polymers.

These are synthetic branched polymer which act as drugs in their own right against HIV (McCarthy et al., 2005). Dendrimers act both as therapeutic agents and non-viral vectors of chemical agents for HIV treatment. Some dendrimers with functional end groups associate with the gp120 of HIV and CD4 molecule of host cell to inhibit the attachment of HIV to the host cell, where some of the dendrimers are capable of interrupting into the cell and restrict with the later stages of HIV replication as well (Peng et al., 2013). Study on polyanionic carbosilane dendrimers G3-S16 and G2-NF16 with sulfated and naphthylsulfonated showed blocking the entry of different X4 and R5 HIV-1. The combinations of G2-STE16 with other carbosilane dendrimers showed a synergistic profile with 100% inhibition against different HIV-1 isolates (Sepulveda-Crespo et al., 2014). Combinations of carbosilane dendrimers/MRV against HIV-1 strains showed synergistic profile (Córdoba et al., 2013). SPL7013 is a dendrimer with broad spectrum activity against both X4 and R5 HIV-1 strains. It prevents viral entry by blocking viral attachment and entry. The polyamidoamine (PAMAM) dendrimer targets the gp120–CD4 complex at two stages: it deteriorates the complex and also modifies its dissociation pathway, potentially inhibiting HIV-1 entry. PAMAM disrupted salt bridges and hydrogen bonds across the gp120–CD4 interface and altered the hydration pattern of the hydrophobic cavity in the interface (Nandy et al., 2013).

### Nanofibers

Various properties of nanofibers make them useful in different fields including its enormous surface area, high porosity, small pore size and the diameter of fibers (Rath et al., 2015, 2016b). Different techniques are available for the synthesis of nanofiber including electrospinning, self-assembly and phase separation (Rath et al., 2016a). Jiang and coworkers prepared pH-responsive drug delivery system by coating the mussel-inspired protein, polydopamine using poly (ɛ-caprolactone) polymer and mediated surface functionalization of electrospun nanofibers. Nanofibers were also used for the treatment of HIV by introducing anti-HIV drugs into vagina. Electrospun fibers of cellulose acetate phthalate (CAP) were prepared for semen induced anti-HIV vaginal drug delivery. Fibers prepared by electrospinning process were incorporated with anti-viral drugs in CAP fibers for pH dependent release of anti-viral drug from fibers in presence of human semen. Cellulose acetate phthalate nanoﬁbers were well tolerated by vaginal epithelial cells and vaginal microflora. Due to pH-sensitive nature of CAP, nanoﬁbers maintained integrity in acidic pH (vaginal environment). However, addition of semen to nanoﬁbers led to immediate dissolution of CAP. CAP nanoﬁbers retained the ability to prevent HIV-1 entry. Incorporation of tenofovir in these nanoﬁbers signiﬁcantly improved its antiviral activity. Polymers like polyvinylpyrrolidone, poly-l-lactide, PLGA, PCL were also used for the fabrication and incorporating different antiviral drugs either individually or as composite.

### Others nanosystems (cyclodextrins, polymeric micelles) (Figure 4)

Cyclodextrins are cyclic oligosaccharides that form hydrophilic inclusion complexes with small or large compounds or can form cyclodextrin drug conjugates (Gaurav et al., 2015a). They can be used in pharmaceutical applications for multifunctional purposes due to its biocompatible nature. Cyclodextrins exhibit property of complexation with different substances which are useful for the enhancement of different properties including increased efficacy, stability and solubility of poorly soluble drugs. Different anti HIV-1 molecules including some nano-metalo herbal complexes were also delivered after forming inclusion complex with different cyclodextrin derivatives (Gaurav et al., 2014, 2015a). Reports also suggest that a derivative cyclodextrin sulfate can block attachment of HIV virions in a nonspecific sense.

**Figure 4.**
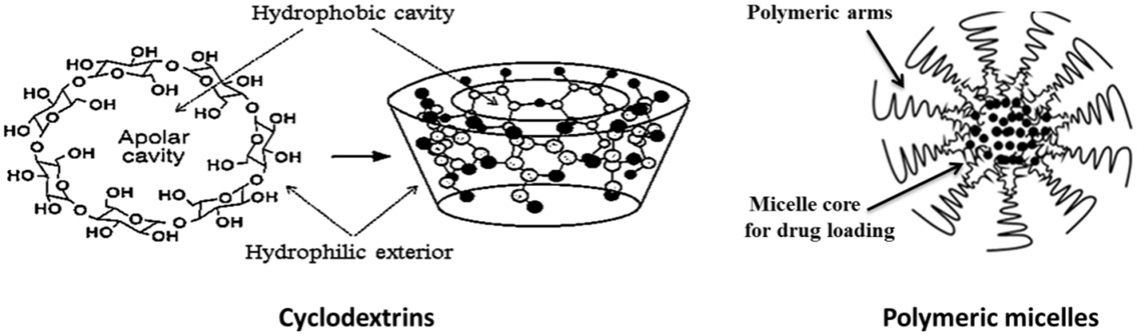
Cyclodextrins and polymeric micelle as nanodrug delivery systems.

Polymeric micelles are a type of nanodelivery systems that hold the poorly aqueous soluble drug in the hydrophobic interior of the micelles and cause drug delivery. They are nanocarriers of nanoscopic shell/core structures formed through amphiphilic block copolymers. Targeted drug delivery, controlled drug release and drug solubilization are their properties that make them suitable for drug delivery purposes. The polymeric micelles showed better results as vaginal microbicide therapy rather than other routes. NanoViricides Inc. is a trademark for polymeric chemical chain which is covalently attached to ligands on virus particles to engulf or coat it for specific virus target. This leads to neutralize the virus infectivity, destabilize and may dismantle it. Thus nano-microbicides are potentially advanced techniques (Aliabadi & Lavasanifar, 2006). Croy et al. reviewed on polymeric micelles for drug delivery to reduce toxicities, delivery to targeted sites and to enhance the therapeutic efficacy of active pharmaceutical ingredients. Polymeric micelles composed of block copolymers have been utilized for improving aqueous solubility, membrane permeability and site-specific delivery of several drug moieties. They have a vesicular or core shell structure similar to surfactant-based micelles, but self-associate at much lower concentrations, typically in the 0.1–1 μM range compared with 0.1–1 mM for surfactant-based micelles. The core of the micelles is usually derived from polymers such as propylene oxide, aspartic acid, l-lysine and caprolactone which constitute the hydrophobic block.

## Conclusion

An efficient anti-HIV treatment will surely have a great impact on the world’s population specifically in the microbicide arena where topical prophylaxis is the key. The widespread requirement of the era where vaccination is not available critically requires a dependable strategy. New class of ARVs and their combination therapeutics provided a positive reflection but no real curable success. Need is there to reassess the potential of the targets we are following till date. Fusion and binding inhibition seems to possess that potential, with the only target which control the viral bioload before it get host entry. Basics of the anatomical and physiological factors related to HIV and its host cells, ease us to understand various available as well as hidden targets. Selection of the delivery system is the key to exploit these targets and the outcomes will be more positive if rational use of nanotechnological approaches would be made.

**Figure 5.**
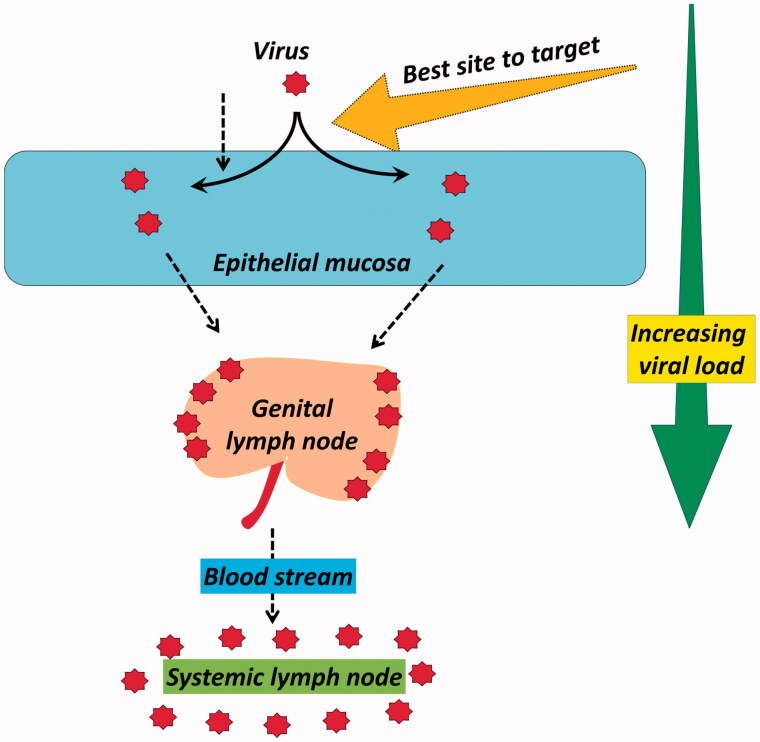
Logical assessment of the best inhibition target with respect to HIV viral load.

Reduced bio-load is always beneficial to tackle any pandemic’s progression and especially in case of HIV where host involvement is the key for its spread. Among the versatile therapeutic strategies to sequester its spread or provide prophylaxis from the existing modes, inhibiting the access of virus inside the host cell seems to be most interesting. Figure 5 attempts to analyze a very simple concept of viral load and the site where an efficient inhibition can be attained. HIV-1 binding and fusion (entry) inhibitors seem to be the fascinating option in this contrast. A number of candidates had gone through the screening with their possible targets of viral attachment, co-receptor binding and fusion. Structural exploration of the receptors and co-receptors is continuously aiding the research units to seek newer targets. Especially for topical pre-exposure prophylaxis (PrEP) where sexual transmission via vaginal and rectal route is under scan, this strategy responds quite early (usually within few minutes to couple of hours). After surfactants and viral disrupting agents which act on the spot of viral exposures this is fastest acting control, while RTI, protease and integrase inhibitors generally take hours to days. Presently, the clinical stake of binding and fusion inhibitors is not up to the mark expressing the negligence of such key target. Moreover the selection of the drug delivery approaches is not need based.

Focusing on the anatomical factors of both HIV and host cells is the key to exploit this fusion inhibition. First challenge in this context is the structural exploration of all the associated receptors and co-receptors. Pharmacoinformatic persons are working in this field to detail the structures and to optimize the pharmacophores of new entities having the capacity to bind these sites. Kinetics and specificity of binding of a new entity with a receptor or co-receptor will determine its potential in real world. Failure of immunization trials literally ends the investment in the area of anti-HIV vaccine development. New vaccination strategies are required aiming the targets responsible for fusion and binding of HIV.

As mentioned in the introduction, lipid composition of the virus is one of the decisive reasons behind the smartness of HIV. Till date, only receptors and co-receptors are targeted for this kind of inhibition. But it is important to understand that it is the flexibility of the viral envelop which regulates most of viral fusion events. Nanotechnology approaches aiming this lipid such as nanocarbon and cyclodextrins which can carry drug too, will be a great solution to strengthen this inhibition approach. Rational selection of drug delivery platform and exploiting the nanosystems can surely improve this approach.

## Supplementary Material

Supplementary_information.docx

## Notes

**Supplementary materials available online.**
