# Supplementary material for: “Fusion and binding inhibition” key target for HIV-1 treatment and pre-exposure prophylaxis: targets, drug delivery and nanotechnology approaches
Source: Drug Deliv. 2017 Feb 26;24(1):608–21. doi: 10.1080/10717544.2016.1228717 (PMC8241151; doi:10.1080/10717544.2016.1228717)
Supplement: Supplementary_information.docx [file IDRD_A_1228717_SM0926.docx]

**Supplementary File**

**“Fusion and Binding Inhibition” Key Target For HIV-1 Treatment and Pre-Exposure-Prophylaxis: Targets, Drug Delivery and Nanotechnology approaches.**

Tanushree Malik**^1^**, Gaurav Chauhan**^1,2^**, Goutam Rath**^1^**, RSR Murthy**^1^**, Amit K Goyal**^1^**

**^1^DBT Lab, Indo Soviet Friendship College of Pharmacy, Moga, Punjab, INDIA.**

**^2^Centre for Nanosciences, Department of Chemical Engineering, Indian Institute of Technology Kanpur, UP, India**

1. **FACTORS RELATED TO VIRUS**

***(Description about HIV-1 membrane, membrane lipids and membrane glycoproteins is detailed in supplementary data).***

**2.1 HIV membrane:** The viral membrane is a lipid bilayer ∼6-10 nm thick, interspersed with membrane-embedded glycoproteins. Viral membrane is derived from host cell membrane with incorporation of some specific host proteins during budding process. A typical HIV viral membrane contains ∼300,000 lipids, with a rather unique distribution of lipids ([Aloia et al., 1988](#_ENREF_1)). Fluidity of viral envelope is low and also highly ordered membrane. The cholesterol to Phospholipids (C/P) molar ratio of membrane of HIV-1 and HIV-2 shown to be 7.5% and 10.5% more ordered than host cell surface membranes ([Aloia et al., 1993](#_ENREF_2)). HIV has a low lipid-to-protein (weight) ratio and a high C/P ratio. So high C/P ratio within viral envelopes has to be required for infectivity ([Slosberg and Montelaro, 1982](#_ENREF_27)).

**2.2 Lipids:** With an average outer HIV-1 diameter of 60-120 nm, yields a theoretical number of 255,000 lipids per particle assuming a 0.5 nm² surface area per lipid molecule ([Briggs et al., 2003](#_ENREF_4)). Lipid composition of HIV-1 is similar to lipid micro domains (rafts) in at least five different aspects i.e. enriched in saturated lipids PS (Phosphotidylserine), pl-PE(plasmenylethanolamine), cholesterol, and sphingolipids ([Pike et al., 2002](#_ENREF_24)). From a study, SM (Sphyngomylein), pl-PE and PS were enriched in viral membranes by a factor of 3.2(SMs), 2.1(PS), and 1.7(pl-PE). PC (Phosphotidylcholine) and PE (Phosphotidyletanolamine) reduced in viral membranes by a factor of 2.7 and 2. DHSM (Dihydrosphingomylein) causes more ordered membranes with a higher melting temperature due to its less polar nature (DHSM is an uncommon raft component and it probably depend form which cell HIV buds) ([Brugger et al., 2006](#_ENREF_5)). Budding of HIV-1 particles occurs at highly specialized membrane microdomains known as lipid rafts ([Hanzal-Bayer and Hancock, 2007](#_ENREF_14)). Lipid Rafts diﬀer in their lipid and protein composition from the surrounding membrane that includes high concentrations of cholesterol, sphingolipids, and glycolipids resulting in a tightly packed DRM(Detergent Resistant Membrane) ([Liao et al., 2003](#_ENREF_19)). HIV budding is a specific raft clustering process, whereas DRM represents general aggregations of raft microdomains ([Brugger et al., 2006](#_ENREF_5)). Head groups of gangliosides in lipid bilayer, particularly sialic acids attached to a lactose group promote DC recognition and to establish infection in the body ([Sedwick, 2012b](#_ENREF_26)).

**2.3 HIV glycoproteins:** The HIV membrane proteins are expressed in the endoplasmic reticulum as a precursor protein, gp160, The precursor is cleaved in the trans-Golgi by the cellular protease furin into two proteins, gp120 and gp41.gp120 binds virus to the cell surface, membrane-spanning gp41 for membrane fusion ([Wyatt and Sodroski, 1998](#_ENREF_30)). These proteins are present on the cell surface as the membrane complex, a mushroom-shaped trimer of heterodimers of gp120 and gp41, incorporated into the viral envelope through the transmembrane region of gp41 as virus particles bud from the cell surface protease (only few envelope glycoprotein spikes are inserted in the viral membrane together with host cell proteins) ([Hallenberger et al., 1992](#_ENREF_13)). The number of gp120/gp41 trimers per virion ranges between 10 and 100 depending on the isolate. HIV gp120 is ∼50% carbohydrate and is one of the most glycosylated proteins known ([Leonard et al., 1990](#_ENREF_18)).

**2.3.1 HIV gp120:** It is an exterior membrane glycoprotein containing 481 amino acids which form spikes sticking out of virus. Each spikes having three non-covalently bound gp120 and gp41 subunits. It contains 5 hyper variable regions from V1 to V5. The structure of gp120 is composed of two mixed α- and β-domains , so-called “inner domain,” which interacts with the gp41 subunit, the larger, less conserved “outer domain,” which is highly glycosylated and interacts with CD4 and the CR proteins of the target cells([Kwong et al., 1998](#_ENREF_17)). It is V3 loop, which determines CR selectivity, the V1/V2 loops determine interface with gp41 and the unbound state in HIV ([Kwon et al., 2012](#_ENREF_16)). The envelope glycoprotein 120 contains 25 beta-strands,5 alpha-helices and 10 defined loop segments. Two conserved regions i.e. bridging beta sheet and V3 loop having conserved base, flexible stem, a beta-hairpin tip.

**2.3.2 HIV gp41:** HIV gp41 is a transmembrane glycoprotein that transverses the lipid bilayer of the virion. It is half embedded in virus membrane, which enables it to bind with gp120.The amino-terminus of gp41, contain a hydrophobic glycine-rich “fusion” peptide that is essential for membrane fusion([Chan et al., 1997](#_ENREF_6)). It can be divided into three major domains: the extracellular domain or ectodomain, the transmembrane domain, and the cytoplasmic domain. The major functions of the gp41 protein are mediated by the extracellular domain, which can be further subdivided into the following five functional regions: the fusion peptide (FP) followed by the N-terminal heptad repeat (NHR), the loop region, the C-terminal heptad repeat (CHR), and finally, the membrane-proximal external region (MPER).Three NHR helices form a coiled-coil core, and the three CHR helices dock in the three hydrophobic grooves around the core, forming the iconic six-helix bundle (6HB) structure ([Garg et al., 2011](#_ENREF_11)).

**4. MODE OF TRANSMISSION**

HIV virions release from infected donor cells interact with epithelial cells and transverse the epithelium through several pathways, including transcytosis, endocytosis, or productive infection, or they merely penetrate through gaps between epithelial cells.([Wu et al., 2003](#_ENREF_29)). Mechanical micro abrasions of the mucosal surface induced by intercourse may allow HIV to directly access target cells ([Bomsel, 1997](#_ENREF_3)). This route might permit HIV-1 to directly contact and then infect intraepithelial Langerhans cells and CD4T cell ([Hladik et al., 2007](#_ENREF_15)).

HIV exposure induces human cervical epithelial cells to produce Thymic Stromal Lymphopoietin (TSLP) cytokine, which activate human myeloid dendritic cells (mDC) and LC, to cause the homeostatic proliferation of autologous CD4+T cells. Interactions of HIV-1 gp120 with transmembrane heparin sulfate proteoglycans and glycoprotein 340, a splice variant of salivary agglutinin expressed by genital epithelial cells, contribute to HIV-1 entry.

Once within the epithelium, HIV encounters CD4+ T cells as well as Langerhans cells ([Nishibu et al., 2006](#_ENREF_22)). HIV-1 are swallowed and stashed in a sac-like compartment within DCs, where they wait to assault new T cells ([Wiley and Gummuluru, 2006](#_ENREF_28)). Mature DCs recognize gangliosides that are present in the HIV’s lipid envelope([Sedwick, 2012a](#_ENREF_25)). A surface protein DC-SIGN, captures HIV-1 retaining it in infectious form presented to permissive CD4-positive T cells([Geijtenbeek et al., 2000](#_ENREF_12)). Increased Siglec-1 expression on the surface of DCs enhanced HIV uptake by mature DCs ([Sedwick, 2012b](#_ENREF_26)). The C-type lectin receptors such as DC-SIGN, mannose receptor, langerin and DCIR interact with virus particles by binding high-mannose oligosaccharides on the heavily glycosylated HIV Env ([Lin et al., 2003](#_ENREF_20)).

Coreceptor engagement; a triggering event that causes the viral fusion protein to undergo conformational changes. On coreceptor triggering, amino-terminal fusion peptide of gp41 exposed and interacts with target cell membrane by formation of a triple-stranded coiled-coil, effectively bridging the two membranes.([Chan et al., 1997](#_ENREF_6)). A chemokine receptor antagonist TAK779, binds to a region of CCR5 and blocks gp120–CCR5 binding and prevents virus infection by altering CCR5 conformation ([Dragic et al., 2000](#_ENREF_10)).

Inside cell viral gene expression in two stages, first regulatory proteins Tat, Rev, and Nef, and secondly the late gene products Gag, Env, Vif, Vpr and Vpu, or Vpx. All involved in some aspect of virion formation. Nef impacts on the replication and survival of HIV in the body([Deacon et al., 1995](#_ENREF_9)). HIV Nef and Vpu also binds cholesterol and delivers it to the cell membrane where new virus particles (called virions) are assembled([Chen et al., 1996](#_ENREF_7)). Cholesterol efﬂux to an extracellular molecule called apoAI was impaired in HIV-infected macrophages with higher levels of viral replication ([Mujawar et al., 2006](#_ENREF_21)). HIV may selectively bud from lipid rafts ([Cosson, 1996](#_ENREF_8)). In polarized cells in the absence of Env, HIV-1 is released from the entire cell surface; the viral glycoprotein restricts this process to the basolateral region ([Owens et al., 1991](#_ENREF_23)).

**REFERENCE**

ALOIA, R. C., JENSEN, F. C., CURTAIN, C. C., MOBLEY, P. W. & GORDON, L. M. 1988. Lipid composition and fluidity of the human immunodeficiency virus. *Proc Natl Acad Sci U S A,* 85**,** 900-4.

ALOIA, R. C., TIAN, H. & JENSEN, F. C. 1993. Lipid composition and fluidity of the human immunodeficiency virus envelope and host cell plasma membranes. *Proceedings of the National Academy of Sciences,* 90**,** 5181-5185.

BOMSEL, M. 1997. Transcytosis of infectious human immunodeficiency virus across a tight human epithelial cell line barrier. *Nat Med,* 3**,** 42-7.

BRIGGS, J. A., WILK, T., WELKER, R., KRAUSSLICH, H. G. & FULLER, S. D. 2003. Structural organization of authentic, mature HIV-1 virions and cores. *EMBO J,* 22**,** 1707-15.

BRUGGER, B., GLASS, B., HABERKANT, P., LEIBRECHT, I., WIELAND, F. T. & KRAUSSLICH, H. G. 2006. The HIV lipidome: a raft with an unusual composition. *Proc Natl Acad Sci U S A,* 103**,** 2641-6.

CHAN, D. C., FASS, D., BERGER, J. M. & KIM, P. S. 1997. Core structure of gp41 from the HIV envelope glycoprotein. *Cell,* 89**,** 263-273.

CHEN, B. K., GANDHI, R. T. & BALTIMORE, D. 1996. CD4 down-modulation during infection of human T cells with human immunodeficiency virus type 1 involves independent activities of vpu, env, and nef. *Journal of virology,* 70**,** 6044-6053.

COSSON, P. 1996. Direct interaction between the envelope and matrix proteins of HIV-1. *The EMBO journal,* 15**,** 5783.

DEACON, N., TSYKIN, A., SOLOMON, A., SMITH, K., LUDFORD-MENTING, M., HOOKER, D., MCPHEE, D., GREENWAY, A., ELLETT, A. & CHATFIELD, C. 1995. Genomic structure of an attenuated quasi species of HIV-1 from a blood transfusion donor and recipients. *Science,* 270**,** 988-991.

DRAGIC, T., TRKOLA, A., THOMPSON, D. A., CORMIER, E. G., KAJUMO, F. A., MAXWELL, E., LIN, S. W., YING, W., SMITH, S. O. & SAKMAR, T. P. 2000. A binding pocket for a small molecule inhibitor of HIV-1 entry within the transmembrane helices of CCR5. *Proceedings of the National Academy of Sciences,* 97**,** 5639-5644.

GARG, H., VIARD, M., JACOBS, A. & BLUMENTHAL, R. 2011. Targeting HIV-1 gp41-induced fusion and pathogenesis for anti-viral therapy. *Curr Top Med Chem,* 11**,** 2947-58.

GEIJTENBEEK, T. B., KWON, D. S., TORENSMA, R., VAN VLIET, S. J., VAN DUIJNHOVEN, G. C., MIDDEL, J., CORNELISSEN, I. L., NOTTET, H. S., KEWALRAMANI, V. N., LITTMAN, D. R., FIGDOR, C. G. & VAN KOOYK, Y. 2000. DC-SIGN, a dendritic cell-specific HIV-1-binding protein that enhances trans-infection of T cells. *Cell,* 100**,** 587-97.

HALLENBERGER, S., BOSCH, V., ANGLIKER, H., SHAW, E., KLENK, H. D. & GARTEN, W. 1992. Inhibition of furin-mediated cleavage activation of HIV-1 glycoprotein gp160. *Nature,* 360**,** 358-61.

HANZAL-BAYER, M. F. & HANCOCK, J. F. 2007. Lipid rafts and membrane traffic. *FEBS letters,* 581**,** 2098-2104.

HLADIK, F., SAKCHALATHORN, P., BALLWEBER, L., LENTZ, G., FIALKOW, M., ESCHENBACH, D. & MCELRATH, M. J. 2007. Initial events in establishing vaginal entry and infection by human immunodeficiency virus type-1. *Immunity,* 26**,** 257-70.

KWON, Y. D., FINZI, A., WU, X., DOGO-ISONAGIE, C., LEE, L. K., MOORE, L. R., SCHMIDT, S. D., STUCKEY, J., YANG, Y., ZHOU, T., ZHU, J., VICIC, D. A., DEBNATH, A. K., SHAPIRO, L., BEWLEY, C. A., MASCOLA, J. R., SODROSKI, J. G. & KWONG, P. D. 2012. Unliganded HIV-1 gp120 core structures assume the CD4-bound conformation with regulation by quaternary interactions and variable loops. *Proc Natl Acad Sci U S A,* 109**,** 5663-8.

KWONG, P. D., WYATT, R., ROBINSON, J., SWEET, R. W., SODROSKI, J. & HENDRICKSON, W. A. 1998. Structure of an HIV gp120 envelope glycoprotein in complex with the CD4 receptor and a neutralizing human antibody. *Nature,* 393**,** 648-59.

LEONARD, C. K., SPELLMAN, M. W., RIDDLE, L., HARRIS, R. J., THOMAS, J. N. & GREGORY, T. J. 1990. Assignment of intrachain disulfide bonds and characterization of potential glycosylation sites of the type 1 recombinant human immunodeficiency virus envelope glycoprotein (gp120) expressed in Chinese hamster ovary cells. *J Biol Chem,* 265**,** 10373-82.

LIAO, Z., GRAHAM, D. R. & HILDRETH, J. E. 2003. Lipid rafts and HIV pathogenesis: virion-associated cholesterol is required for fusion and infection of susceptible cells. *AIDS research and human retroviruses,* 19**,** 675-687.

LIN, P.-F., BLAIR, W., WANG, T., SPICER, T., GUO, Q., ZHOU, N., GONG, Y.-F., WANG, H.-G. H., ROSE, R. & YAMANAKA, G. 2003. A small molecule HIV-1 inhibitor that targets the HIV-1 envelope and inhibits CD4 receptor binding. *Proceedings of the National Academy of Sciences,* 100**,** 11013-11018.

MUJAWAR, Z., ROSE, H., MORROW, M. P., PUSHKARSKY, T., DUBROVSKY, L., MUKHAMEDOVA, N., FU, Y., DART, A., ORENSTEIN, J. M. & BOBRYSHEV, Y. V. 2006. Human immunodeficiency virus impairs reverse cholesterol transport from macrophages. *PLoS biology,* 4**,** e365.

NISHIBU, A., WARD, B. R., JESTER, J. V., PLOEGH, H. L., BOES, M. & TAKASHIMA, A. 2006. Behavioral responses of epidermal Langerhans cells in situ to local pathological stimuli. *J Invest Dermatol,* 126**,** 787-96.

OWENS, R. J., DUBAY, J. W., HUNTER, E. & COMPANS, R. W. 1991. Human immunodeficiency virus envelope protein determines the site of virus release in polarized epithelial cells. *Proceedings of the National Academy of Sciences,* 88**,** 3987-3991.

PIKE, L. J., HAN, X., CHUNG, K. N. & GROSS, R. W. 2002. Lipid rafts are enriched in arachidonic acid and plasmenylethanolamine and their composition is independent of caveolin-1 expression: a quantitative electrospray ionization/mass spectrometric analysis. *Biochemistry,* 41**,** 2075-88.

SEDWICK, C. 2012a. How HIV sneaks aboard mature dendritic cells. *PLoS Biol,* 10**,** e1001454.

SEDWICK, C. 2012b. Lipids in HIV's envelope help the virus to spread. *PLoS Biol,* 10**,** e1001316.

SLOSBERG, B. N. & MONTELARO, R. C. 1982. A comparison of the mobilities and thermal transitions of retrovirus lipid envelopes and host cell plasma membranes by electron spin resonance spectroscopy. *Biochim Biophys Acta,* 689**,** 393-402.

WILEY, R. D. & GUMMULURU, S. 2006. Immature dendritic cell-derived exosomes can mediate HIV-1 trans infection. *Proc Natl Acad Sci U S A,* 103**,** 738-43.

WU, Z., CHEN, Z. & PHILLIPS, D. M. 2003. Human genital epithelial cells capture cell-free human immunodeficiency virus type 1 and transmit the virus to CD4+ Cells: implications for mechanisms of sexual transmission. *J Infect Dis,* 188**,** 1473-82.

WYATT, R. & SODROSKI, J. 1998. The HIV-1 envelope glycoproteins: fusogens, antigens, and immunogens. *Science,* 280**,** 1884-8.
